# Isothermal Amplification and CRISPR/Cas12a-System-Based Assay for Rapid, Sensitive and Visual Detection of *Staphylococcus aureus*

**DOI:** 10.3390/foods12244432

**Published:** 2023-12-11

**Authors:** Danhong Xu, Haijuan Zeng, Wenhui Wu, Hua Liu, Jinbin Wang

**Affiliations:** 1 School of Food Science and Technology, Shanghai Ocean University, Shanghai 201499, China; xudanhong2023@163.com (D.X.); whwu@shou.edu.cn (W.W.); 2 Key Laboratory of Agricultural Genetics and Breeding, The Biotechnology Research Institute, Shanghai Academy of Agricultural Sciences, Shanghai 201106, China; zenghaijuan12@126.com (H.Z.); liuhua0212@foxmail.com (H.L.); 3 Crops Ecological Environment Security Inspection and Supervision Center, Ministry of Agriculture and Rural Affairs, Shanghai 201106, China

**Keywords:** *S. aureus*, LAMP, RPA, immunochromatographic test strip, fluorescence detection

## Abstract

*Staphylococcus aureus* exists widely in the natural environment and is one of the main food-borne pathogenic microorganisms causing human bacteremia. For safe food management, a rapid, high-specificity, sensitive method for the detection of *S. aureus* should be developed. In this study, a platform for detecting *S. aureus* (*nuc* gene) based on isothermal amplification (loop-mediated isothermal amplification—LAMP, recombinase polymerase amplification—RPA) and the clustered regularly interspaced short palindromic repeats (CRISPR) and CRISPR-associated (Cas12a) proteins system (LAMP, RPA-CRISPR/Cas12a) was proposed. In this study, the LAMP, RPA-CRISPR/Cas12a detection platform and immunochromatographic test strip (ICS) were combined to achieve a low-cost, simple and visualized detection of *S. aureus*. The limit of visual detection was 57.8 fg/µL of *nuc* DNA and 6.7 × 10^2^ CFU/mL of bacteria. Moreover, the platform could be combined with fluorescence detection, namely LAMP, RPA-CRISPR/Cas12a-flu, to establish a rapid and highly sensitive method for the detection of *S. aureus*. The limit of fluorescence detection was 5.78 fg/µL of genomic DNA and 67 CFU/mL of *S. aureus*. In addition, this detection platform can detect *S. aureus* in dairy products, and the detection time was ~40 min. Consequently, the isothermal amplification CRISPR/Cas12a platform is a useful tool for the rapid and sensitive detection of *S. aureus* in food.

## 1. Introduction

*Staphylococcus aureus* [1], one of the top five foodborne pathogens, has a common and strong aggressiveness in humans and can secrete multiple toxic proteins (Pathogenic enterotoxins, Hemolysin, PVL) [1,2] which can cause bacteraemia, endocarditis, meningitis, toxic shock syndrome, pneumonia and other dangerous infectious diseases [3]. Moreover, the worldwide annual incidence rate of bacteremia is 15–40 cases per 100,000 people [4]. *S. aureus* bacteraemia is associated with a mortality rate of about 15–25% [4]. *S. aureus* is widely found in a variety of environments, such as air, water, livestock, poultry and their waste, making food vulnerable to contamination [5], and in the United States, there are approximately 241,000 cases of foodborne illness caused by *S. aureus* each year [6]. Therefore, the possibility of the direct or indirect contamination of food with *S. aureus* increases, greatly increasing the risk of infections.

Traditional detection methods, such as culture-based counting for colony-forming units, heterotrophic plate counts, the spectrophotometer method of optical density measurement and flow cytometry, are simple but need bacteria enrichment and to prolong detection time [7]. Immunoassay methods, for instance, the enzyme-linked immunosorbent assay, enzyme-linked fluoroimmunoassay and immunomagnetic bead separation, are easy to automate but have a degree of complicated operation and long incubation time [8,9,10]. Contrarily, the immunochromatographic test strip (ICS) has been widely used as a rapid, simple and low-cost correct detection technology [11]. Recently, biosensors based on electrochemistry, matrix-assisted laser desorption ionization time-of-flight [12] and surface-enhanced Raman scattering have attracted widespread attention [13]. However, these methods usually require skilled operators and expensive, large-scale instruments, which are not conducive to point-of-care testing (POCT).

Molecular methods, such as multiple polymerase chain reaction (PCR) [14,15] and real-time PCR [15,16,17,18], have also been used for bacterial quantification and identification, but PCR- and RT-PCR-based detection methods require sophisticated thermal cyclers and are time-consuming. In recent years, the isothermal amplification technique (e.g., loop-mediated isothermal amplification, LAMP; recombinase polymerase amplification, RPA) has developed rapidly because of its advantages of high sensitivity, specificity, convenience and simple laboratory equipment, even if water baths or heating blocks are sufficient to provide the essential conditions [19,20,21]. However, the isothermal amplification technique used alone has a high susceptibility to cause false-positive amplification, due to cross-contamination, nonspecific amplification or primer dimerization [22].

The CRISPR/Cas (clustered regularly interspaced short palindromic repeats and CRISPR-associated proteins) is an adaptive immunity system in bacteria and archaea [23]. Cas12a is a class 2 CRISPR-Cas system [24], and Cas12a enzymes recognize a T-nucleotide-rich protospacer-adjacent motif, catalyze their own guide CRISPR RNA (crRNA) maturation and generate a PAM-distal dsDNA break with staggered 5′ and 3′ ends, thus having attracted interest for gene-editing applications like in zebrafish [25,26]. Until 2018, Li et al. confirmed that the trans-cleavage activities and target DNA binding also induced the cleavage of non-target DNA of Cas12a, and they developed HOLMES, which can be used for the fast detection of target DNA [27]. To improve the sensitivity of HOLMES, Cheng et al. combined HOLMES with PCR, and the detectable concentration could reached 10 aM [28].

At present, researchers have combined CRISPR/Cas12a and nucleic acid amplification for the detection of various viruses and microbes. Chaijarasphong et al. coupled a CRISPR-Cas12a fluorescence assay with RPA for the detection of white spot syndrome virus in shrimp [29]. Lee et al. established a detection platform combining a LAMP isothermal amplification system and CRISPR/Cas12a system for the detection of *Escherichia coli* O157:H7 [20]. CRISPR/Cas12a has the potential for rapid, low-cost and non-toxic sequencing of targeted nucleic acids.

In this study, a highly sensitive, visual, rapid and specific sensing strategy named LAMP/RPA-CRISPR/Cas12a-ICS and -flu for detecting *S. aureus* (*nuc* gene) was developed. The strategy integrated isothermal amplification methods (RPA, LAMP) with the CRISPR/Cas12a system to eliminate false positives generated by isothermal amplification. We combined LAMP/RPA-CRISPR/Cas12a with the ICS to achieve the visual and fast detection of *S. aureus*, and also combined this with fluorescence detection, namely LAMP, RPA-CRISPR/Cas12a-flu, to achieve the high-sensitivity detection of *S. aureus*.

## 2. Material and Methods

### 2.1. Materials

The bacteria involved in this study include two species of *S. aureus* and eleven other common foodborne pathogens, as shown in Table S1. The primers, crRNA and ssDNA were synthesized by Sangon (Shanghai, China). The TIANamp Bacteria DNA Kit model DP302, WarmStart LAMP Kit model E1700S, RPA assay kits, rNase Inhibitor and LbCpf1 Nuclease were acquired from Tiangen (Beijing, China), New England Biolabs (Ipswich, MA, USA), TwistDx (Cambridge, MA, USA), Tolo (Shanghai, China), Bersee (Beijing, China) and BBI (Shanghai, China), respectively. Biotin ligand-streptavidin, goat anti-rabbit antibody, BSA and anti-FITC antibody (15 nm gold-nanoparticle-labeled) were purchased from Beijing Bersee (Beijing, China), Merck (Shanghai, China), Beijing Biosynthesis Biotechnology (Beijing, China) and Beijing Solarbio (Beijing, China), respectively. Nitrocellulose (NC) membrane (CN95 and CN140), cellulose fiber (absorbent pads), fiberglass mat (sample pads and conjugate pads) and PVC backing plates were purchased from Merck Chemicals (Shanghai, China). The chemicals and solvents involved in this paper were analytical-grade. A total of 24 samples were included in this study. Four samples (milk, modulated milk, orange juice and soymilk) were purchased at the local supermarket (Ru Hai, Shanghai, China). Information on the remaining 20 natural samples is listed in Table S2.

### 2.2. Genomic DNA Extraction and Primer Design

All bacteria were cultured overnight and the cultivation conditions are shown in Table S1. Genomic DNA extracted from TIANamp Bacteria DNA Kit model DP302 was used for the detection of the *nuc* gene due to its high purity. The extracted genome concentration was determined using NanoDrop 2000c (Thermo Fisher Scientific 5225 Verona Rd Madison, WI53711 Assembled in USA, Waltham, MA, USA) and stored at −20 °C for future use. Bacterial DNA extraction Mini Kit (Mabio, Guangzhou, China) was used to extract genomic DNA for the detection of *S. aureus* due to its fast extraction speed.

Whole-genome sequences of 32 *S. aureus* were retrieved from the National Center for Biotechnology Information (NCBI) to mine specific targets for *S. aureus* (names and accession numbers of 32 strains of *S. aureus* are shown in Table S3). Through local BLASTN comparison, a specific gene (*nuc* GenBank: DQ507382.1) to detect *S. aureus* was obtained. The RPA and PCR primers used in this study were designed from Primer Premier 5.0 (Premier Biosoft, San Francisco, CA, USA, Toronto, ON, Canada). And the LAMP primers and crRNA were designed through the website (http://primerexplorer.jp/ (accessed on 5 January 2022), CRISPOR (tefor.net)). All the detailed information is shown in Table S4.

### 2.3. Construction of ICS

**Figure 1:**
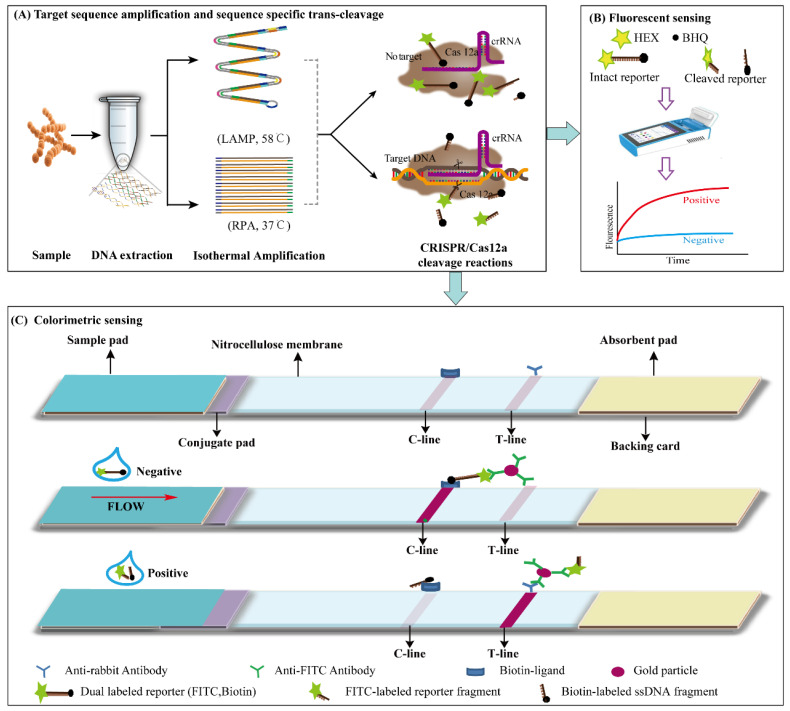
Schematic of CRISPR/Cas12a system combined with isothermal amplification for rapid and visual nucleic acid detection.

The composition of ICS is shown in Figure 1. In detail, the gold nanoparticle (AuNP)-labeled anti-FITC antibodies (2 µg) were stored in the conjugate pad, and the AuNPs were used for coloration. The BioDot-XYZ3060 point drug system (Bio-Dot, Irvine, CA, USA) was used to distribute 2 mg/mL streptavidin and 1 mg/mL goat anti-rabbit IgG antibody on the NC filter membrane in a volume of 3 µL to form control lines (C-line) and test lines (T-lines). The NC filter membrane, coupling pad, sample pad and absorption pad were assembled in sequence on the plastic backplane overlapping each other by 2 mm. Finally, the ICS was cut into 3 mm wide strips using the CM4000 cutter (Bio-Dot, Irvine, CA, USA).

### 2.4. LAMP, RPA and PCR Assay for S. aureus

For LAMP reaction, 12.5 µL 2 × Master Mix, 2.5 µL 10 × LAMP primers, 8 µL ddH_2_O and 2 µL DNA template were added successively and mixed evenly. The reaction condition was incubated at 62.7 °C for 30 min.

The RPA process was performed according to the RPA kit’s instructions. Firstly, 29.5 µL Buffer was mixed with 11.2 µL ddH_2_O and 2.4 µL primer to completely dissolve the enzyme powder. Then, 2 µL DNA template was added and mixed thoroughly. Finally, 2.5 µL MgOAc was added to start the reaction and incubated at 37.7 °C for 15 min. LAMP and RPA results were observed using agarose gel electrophoresis using DL2000 Marker.

PCR amplification was carried out in a 20 μL reaction system, containing 2 μL of template DNA, 1 μL of each primer (10 nM), 12.5 μL of 2 × Taq mix (Sangon Biotech; Shanghai, China), 3.5 μL ddH_2_O. PCR analysis was performed using the following procedures: initial denaturation at 95 °C for 5 min, followed by 35 cycles of denaturation at 95 °C for 10 s, annealing at 58 °C for 30 s and extension at 72 °C for 20 s, with a final extension at 72 °C for 5 min.

### 2.5. LAMP, RPA-CRISPR/Cas12a-ICS and the LAMP, RPA-CRISPR/Cas12a-Flu Assay

The CRISPR/Cas12a reaction system consisted of 50 nM LbCpf1 nucleases, 10 U RNase inhibitor, 500 nM crRNA, 500 nM ssDNA reporter, 2 µL NE buffer, 2 µL DNA template in a 20 µL reaction volume. The DNA template was the LAMP or RPA product, and the ssDNA was a non-targeting probe. The ssDNA reporter of ICS was biotin- and FITC-double-labeled (5′-Biotin-TTATT-FITC-3′), and flu-ssDNA reporter (5′-HEX-10T-BHQ1-3′) was designed for fluorescence detection. Then, the reaction was incubated at 37 °C for 15 min.

For LAMP, RPA-CRISPR/Cas12a-ICS analysis, 5 µL of the cleavage product was loaded to the sample pad, and the ICS was then placed in phosphate-buffered solution, according to the direction indicated for 5 min. Then, the results were directly obtained through visual detection. The fluorescence intensity of ssDNA cleavage reaction products was analyzed using LAMP and RPA-CRISPR/Cas12a-flu. The relative fluorescence intensity was measured using the multi-purpose microplate reader (Tecan, Mannedorf, Switzerland), with excitation and emission wavelengths of 530 nm and 560 nm, respectively. The detection process was described in our previous report [30].

### 2.6. Optimization of Reaction Conditions for LAMP and RPA Based on the CRISPR/Cas12a-Flu Platform

The temperature and time of LAMP and RPA reactions were optimized using the CRISPR/Cas12a-flu platform. The temperature of LAMP-CRISPR/Cas12a platform was optimized at 58~70 °C for 60 min, and then the time was optimized from 0 to ~60 min at the optimum temperature. Similarly, the temperature of RPA-CRISPR/Cas12a platform was optimized at 37~48 °C for 20 min, and then the time was optimized from ~15 to ~30 min at the optimum temperature. The genome concentration used was 10^6^ fg/µL.

### 2.7. Specificity and Sensitivity of LAMP, RPA-CRISPR/Cas12a Platform

The specificity of the LAMP, RPA-CRISPR/Cas12a platform was determined through the detection of the DNA template of two *S. spp*. (*S. xylosus*; *S. mimicus*) and nine non-*S. spp*. strains (Table S1) to assess.

To evaluate the genomic sensitivity and bacterial sensitivity of LAMP, RPA-CRISPR/Cas12a platforms, the genomic DNA and *S. aureus* were diluted tenfold to a concentration of 5.8 × 10^6^ fg/µL to 5.8 fg/µL (equivalent to a molar concentration of 3.2 × 10^5^ fM to 0.32 fM) and 6.7 × 10^6^ CFU/mL to 6.7 CFU/mL respectively. DNase/RNase-free distilled water was used as a non-template control.

### 2.8. Sample Validation

The Chinese official national food safety standard GB29921-2021 [31]sets the limit of detection (LOD) for *S. aureus* at 10^3^ CFU/mL. A lower concentration (10^2^ CFU/mL) was added to samples (milk, reconstituted milk, orange juice and soya milk). As the matrix in the actual samples had an impact on the extraction of *S. aureus* DNA, the culture method in GB 4789.10-2010 [32] was used to incubate the strains at 37 °C for 6 h. After culture and extraction, the concentration of *S. aureus* was 10^5^ CFU/mL, and the concentrations of the *S. aureus* genome in milk, reconstituted milk, orange juice and soya milk were 15 ng/µL, 10 ng/µL, 4 ng/µL and 9 ng/µL, respectively.

Then, DNA of samples was extracted using the previously described Alkaline Lysis (AL) method [33]. Briefly, one part of sample (1 mL) was collected through centrifugation (12,000× *g* for 2 min) and resuspended in 25 µL of sterile distilled water. Then, 25 µL of 50 mM NaOH was added to the bacterial suspension before it was incubated at 100 °C for 10 min. After neutralization with 4 µL of 1 M Tris-HCl buffer (pH 7.5), the supernatant was used as template DNA.

### 2.9. Statistical Analysis

The data treatment was consistent with our previous study [34]. Briefly, data analysis and graphing were performed using SPSS 23.0 (IBM Crop., Chicago, IL, USA) and Origin 2021 (Northampton, MA, USA). Each result was obtained using the mean ± SD of at least three independent experiments.

## 3. Results

### 3.1. Principle of LAMP, RPA-CRISPR/Cas12a Platform

To detect *S. aureus* in food quickly, specifically and sensitively, a LAMP, RPA-CRISPR/Cas12a detector platform was successfully established by combining ICS and fluorescence detection. The detection process of the LAMP, RPA-CRISPR/Cas12a platform is shown in Figure 1. Four steps were used to detect *S. aureus*, including the (i) extraction of *S. aureus* genomic DNA from the sample mixture, (ii) amplification of *S. aureus* genomic DNA using LAMP or RPA, (iii) identification and cleavage of the target gene *nuc* using the CRISPR/Cas12a system and (iv) release signals for ICS or fluorescence detection.

When the crRNA recognizes the amplified target sequence, the trans-cleavage activity of the binary complex was stimulated, and the ssDNA reporter genes in the reaction solution was cleaved to release the detection signal. In the LAMP, RPA-CRISPR/Cas12a-flu platform, the quencher BHQ1 was separated from the fluorochrome HEX, resulting in the production of fluorescence signals. Without target sequences, *trans* cleavage was inactive, and no fluorescence signal was produced. In the LAMP, RPA-CRISPR/Cas12a-ICS platform, when trans-cleavage was activated, the ICS-ssDNA reporter was cleaved to produce two ssDNA fragments labeled by biotin and FITC, respectively. Then, the ssDNA fragments labeled by FITC were combined with gold nanoparticle (AuNP)-labeled anti-FITC antibodies and formed a new complex on the conjugate pad. With the capillary action, the complex moved to the T-line and combined with the goat anti-mouse IgG antibody, making the T-line red.

### 3.2. Primer Screening of LAMP, RPA-CRISPR/Cas12a Platform

**Figure 2:**
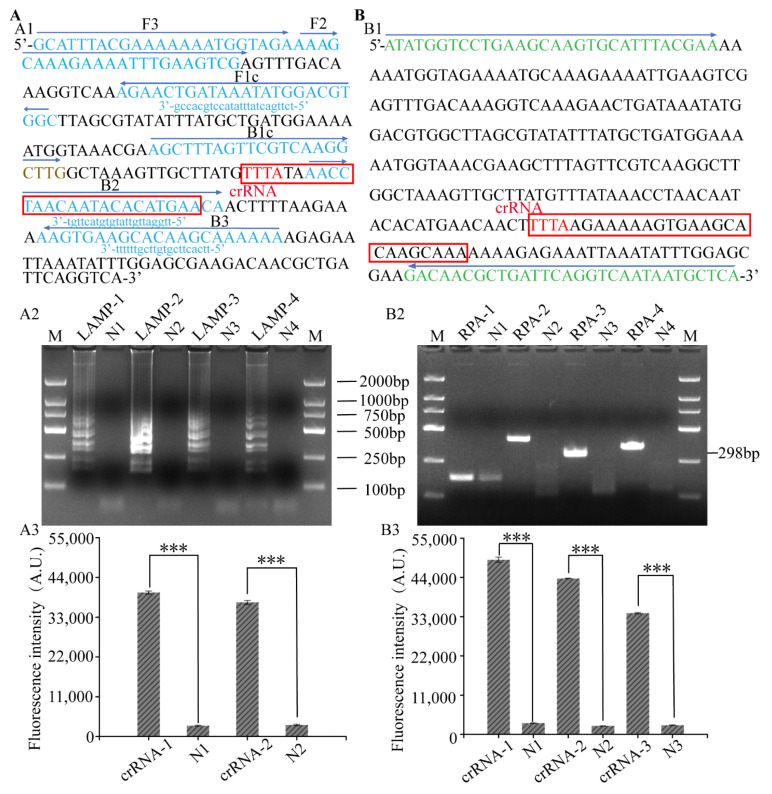
The screening of primers and the crRNA used for the LAMP and RPA reactions. (**A1**,**B1**) Amplified fragments and primers of LAMP and RRA. (**A2**,**B2**) Visualization of amplification products of different primers for LAMP and RPA by agarose gel electrophoresis. (**A3**,**B3**) Relative fluorescence intensity of LAMP-CRISPR/Cas12a and RPA-CRISPR/Cas12a with different crRNA involvements. ***, *p* < 0.001;, with three biological replicates. M, DL2000 Marker; N, no template added as negative control. Red box, crRNA sequences.

To improve the sensitivity of the LAMP, RPA-CRISPR/Cas12a platform, four LAMP and RPA primers were designed for screening. The LAMP-2 primers showed strong target bands and the highest amplification efficiency (Figure 2(A1)). The RPA-3 (Figure 2(B1)) primers showed clear bands and no primer dimerization of the negative control. Two and three crRNAs were separately designed for LAMP and RPA to provide cleavage efficiency for the LAMP, RPA-CRISPR/Cas12a platform. The fluorescence values and ratios of the designed crRNA and the negative control were measured using the microplate reader and are listed in Table S5. crRNA-1 (Figure 2(A2)) and crRNA-2 (Figure 2(A3)) with the highest proportion were selected for subsequent experiments.

### 3.3. Optimization of LAMP and RPA Reaction Conditions for LAMP-CRISPR/Cas12a System

**Figure 3:**
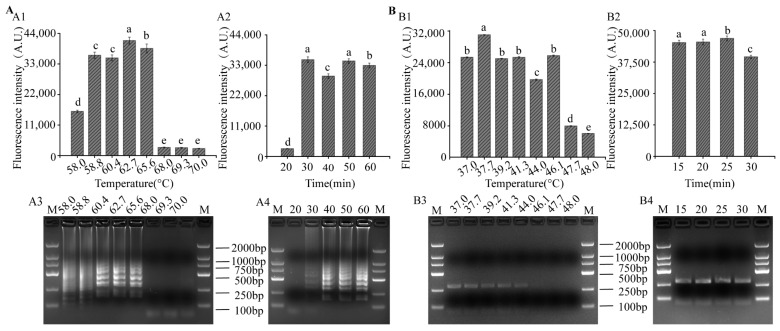
Optimization of LAMP and RPA reaction. (**A1**,**B1**) and (**A2**,**B2**) Relative fluorescence intensity of LAMP-CRISPR/Cas12a and RPA-CRISPR/Cas12a, respectively. (**A3**,**B3**) and (**A4**,**B4**) Visualized detection of LAMP and RPA products by gel electrophoresis, respectively. Different letters indicate that the variables are significantly different (*p* < 0.05); M, DL2000 Marker.

Temperature optimization results of LAMP showed that the relative fluorescence intensity was the highest at 62.7 °C (Figure 3(A1)), which was consistent with the results of gel electrophoresis (Figure 3(A3)). The time optimization results showed that the relative fluorescence intensity was highest at 30 min (Figure 3(A2)) and, at the same time, bands appeared in the gel electrophoresis. In order to shorten the detection time, the optimal LAMP conditions were determined as 62.7 °C for 30 min. When the amplification temperature of RPA was 37.7 °C, the fluorescence intensity was highest (Figure 3(B1)), and the band in gel electrophoresis (Figure 3(B3)) was the brightest. After reacting for 15 min, strong fluorescence signals (Figure 3(B2)) and bands (Figure 3(B4)) appeared; thus, the RPA reaction condition was 37.7 °C for 15 min.

### 3.4. Specificity of LAMP, RPA-CRISPR/Cas12a Platform

**Figure 4:**
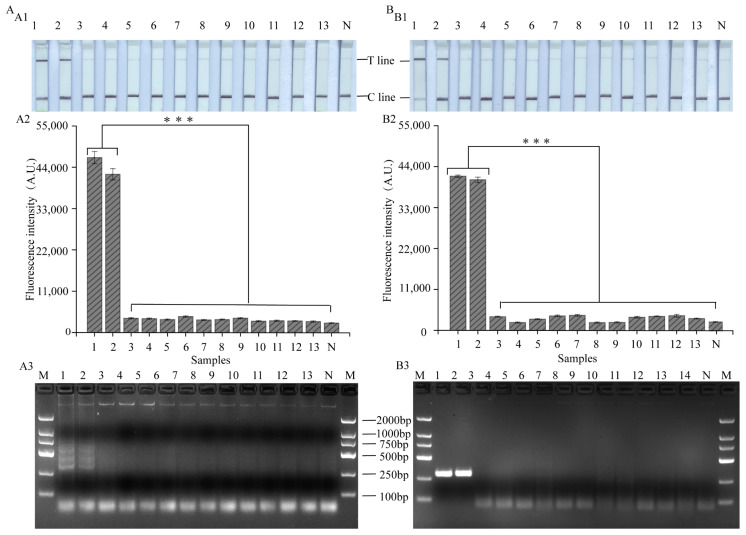
The detection specificity results of the LAMP, RPA-CRISPR/Cas12a platform. (**A1**,**B1**) LAMP- and RPA-based CRISPR/Cas12a-ICS assay for bacteria. (**A2**,**B2**) LAMP- and RPA-based CRISPR/Cas12a-flu assay for bacteria (with three biological replicates). (**A3**,**B3**) Visualized detection of LAMP- and RPA-specific products by gel electrophoresis. (***, *p* < 0.001). Lanes 1–2, positive samples of *S. aureus* ATCC6538, ATCC43300. Lanes 3–13, negative samples of *S. xylosus* ATCC29971, *S. mimicus* ATCC27851, *E. coli.* O157, H7 ATCC43888, ATCC43895, *E. coli.* ATCC25922, ATCC8739, ATCC9673, *V. parahaemolyticus* ATCC33847, ATCC17802, *V. vulnificus* ATCC27562, *V. algolyticus* ATCC33787. N, no template added as negative control. No. 1–13 corresponds to the bacteria in Table S1.

To ensure the specificity of the LAMP, RPA-CRISPR/Cas12a platform, eleven strains, including two *S. spp* (*S. xylosus*; *S. mimicus*) and nine non-*S. spp.* strains, are presented in Table S1. In fact, the T-line displays a strong signal only when the target strain genome is present. Strains without *nuc* genes display weak bands on the T-line, which can produce significant differences from strains with *nuc* genes on the T-line (Figure 4(A1,B1)). In addition, in order to quantitatively analyze the results, ImageJ (NIH, Bethesda, MD, USA) analysis was performed on the detection-specific results of LAMP, RPA-CRISPR/Cas12a-ICS platforms (Figure S1). Similarly, as shown in Figure 4(A2,B2), only the target strain had a strong fluorescent signal (*p* < 0.05), which was consistent with the electrophoretic results (Figure 4(A3,B3)). Thus, the LAMP, RPA-CRISPR/Cas12a platform exhibited acceptable specificity.

### 3.5. Sensitivity of LAMP, RPA-CRISPR/Cas12a Platform

**Figure 5:**
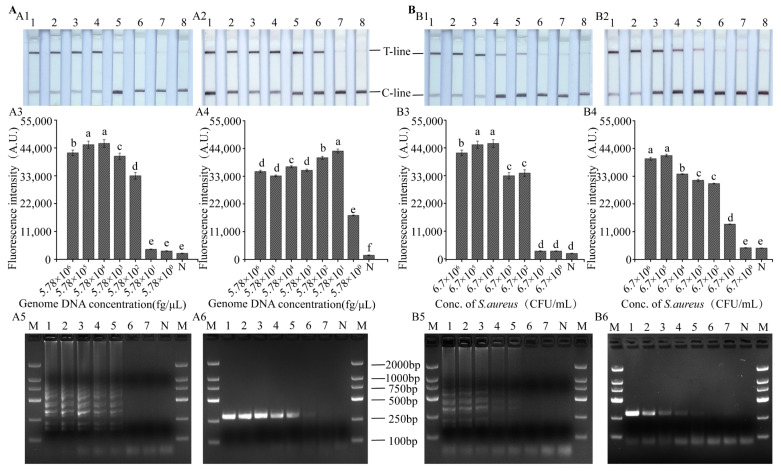
(**A**,**B**) Sensitivity assessment of various detection methods for *nuc* genomic DNA and *S. aureus* strain pure cultures. (**A1**,**B1**) and (**A2**,**B2**) Sensitivity of the LAMP-CRISPR/Cas12a-ICS and RPA-CRISPR/Cas12a-ICS assay for *S. aureus* with optimized reaction conditions viewed by the naked eye. (**A3**,**B3**) and (**A4**,**B4**) Fluorescence results of LAMP-CRISPR/Cas12a-flu and RPA-CRISPR/Cas12a-flu detection method. (**A5**,**B5**) and (**A6**,**B6**) LAMP and RPA products were visualized using an agarose gel electrophoresis platform with a microplate reader (three biological replicates). Different letters indicate that the variables are significantly different (*p* < 0.05). N, no template added as negative control.

To detect the sensitivity, the *nuc* gene at levels from 5.78 × 10^6^ fg/µL to 5.78 fg/µL (3.2 × 10^5^ fM to 0.32 fM) and *S. aureus* at levels from 6.7 × 10^6^ CFU/mL to 6.7 CFU/mL were prepared. LODs of the *nuc* gene detected using the LAMP-CRISPR/Cas12a-ICS (Figure 5(A1)) and LAMP-CRISPR/Cas12a-flu (Figure 5(A3)) platforms and gel electrophoresis (Figure 5(A5)) were all 5.78 × 10^2^ fg/µL. The RPA-CRISPR/Cas12a-ICS platform had an LOD of 57.8 fg/µL for the detection of the *nuc* gene (Figure 5(A2)), which was consistent with the gel electrophoretic results (Figure 5(A6)). However, compared with RPA-CRISPR/Cas12a-ICS and gel electrophoretic methods, the RPA-CRISPR/Cas12a-flu platform had a higher LOD of 5.78 fg/µL (Figure 5(A4)).

Consistent with the genomic sensitivity results, the LAMP-CRISPR/Cas12a-ICS (Figure 5(B1)) and LAMP-CRISPR/Cas12a-flu (Figure 5(B3)) platforms and gel electrophoresis (Figure 5(B5)) had the same LOD of 6.7 × 10^2^ CFU/mL for the detection of *S. aureus*. And the RPA-CRISPR/Cas12a-ICS (Figure 5(B2)) platform and gel electrophoresis (Figure 5(B6)) had an LOD of 6.7 × 10^2^ CFU/mL for the detection of *S. aureus*. However, the RPA-CRISPR/Cas12a-flu (Figure 5(A3)) platform had a higher LOD of 67 CFU/mL (Figure 5(B4)).

### 3.6. Evaluation of the Practical Value of LAMP, RPA-CRISPR/Cas12a Platform

**Figure 6:**
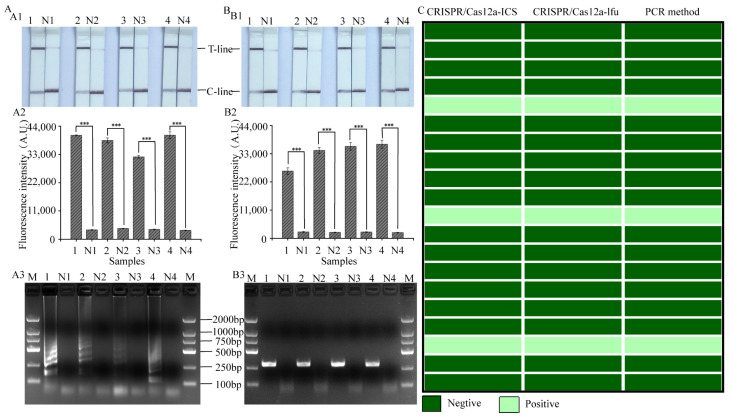
The applicability of the LAMP, RPA-CRISPR/Cas12a platform to detect *S. aureus* in spiked samples and natural samples. (**A1**,**B1**) The LAMP, RPA-CRISPR/Cas12a-ICS assay on different spiked samples viewed by the naked eye. (**A2**,**B2**) The LAMP, RPA-CRISPR/Cas12a-flu assays on different spiked samples. (**A3**,**B3**) LAMP and RPA products from different spiked samples were visualized using agarose gel electrophoresis. (**C**) Comparison of *S. aureus* test results for matching natural samples analyzed using the LAMP-CRISPR/Cas12a-ICS, RPA-CRISPR/Cas12a-flu or PCR method (three biological replicates) (***, *p* < 0.001). 1, milk; 2, reconstituted milk; 3, orange juice; 4, soya milk; N, no template added as negative control.

To evaluate the accuracy and reliability of LAMP and RPA-CRISPR/Cas12a platforms in practical applications, samples contaminated with *S. aureus* at different concentrations and 20 natural samples were detected. The results showed that LAMP, RPA-CRISPR/Cas12a-ICS (Figure 6(A2,B2)) and LAMP, RPA-CRISPR/Cas12a-flu (Figure 6(A3,B3)) platforms were able to detect pathogenic *S. aureus*, which was consistent with the results of gel electrophoresis (Figure 6(A1,B1)). Thus, our developed LAMP, RPA-CRISPR/Cas12a platform could be applied to identify the possible contamination by *S. aureus* in different foods. Among the 20 real samples, three samples were found to be contaminated by *S. aureus*. The detection results (Figure 6C) had a 100% consistency between our assay and the PCR method, indicating that the LAMP, RPA-CRISPR/Cas12a platform had high accuracy.

## 4. Discussions

The detection of *S. aureus* in food is essential to ensure food safety, which helps people avoid the occurrence of foodborne illnesses caused by the consumption of food contaminated with this bacterium [35]. Nucleic-acid-based assays such as PCR, real-time PCR and ddPCR can identify ingredients with high sensitivity but require bulky instrumentation and long assay times [36]. While LAMP and RPA are isothermal technologies that require only minimum laboratorial support and can be easily employed on-site, in this study, the constant-temperature amplification technology was used to amplify the fragment, which reduced the detection time and instrument cost. The LAMP, RPA-CRISPR/Cas12a system was combined with a lateral flow biosensor and fluorescence intensity detection to form the LAMP, RPA-CRISPR/Cas12a-ICS platform and the LAMP, RPA-CRISPR/Cas12a-flu platform for the detection of *S. aureus*. The specific amplification of target DNA by LAMP or RPA and the trans-cleavage activity of activated Cas12a/crRNA greatly enhanced the detection signal [37,38].

Studies have shown that false positive results for negative controls are often observed when the LAMP takes more than 30 min. And isothermal amplification such as LAMP and RPA are prone to non-specific amplification. However, the CRISPR/Cas12a system, which is sequence-specific, can identify amplification products in the region with continuous PAM sites, and then distinguish between positive and false-positive results, providing higher specificity [20].

In this study, two detection systems of the LAMP, RPA-CRISPR/Cas12a detection platform for *S. aureus* have been established with high specificity, which can exclude the interference of complex matrices. In general, the detection limit for RPA-CRISPR/Cas12a was higher than that for LAMP-CRISPR/Cas12a, which may be due to the fact that there were four primers in the LAMP, and the conditions for binding targets were relatively high. Among them, fluorescence detection was used, and the LOD (5.78 fg/μL) reached in this study is 10^3^ times higher than that in the method of Cao et al. (10^3^ fg/μL) [38]. In addition, the sensitivity of *S. aureus* was also studied with an LOD of 67 CFU/mL, which had not been performed before [39,40]. The LAMP-CRISPR/Cas12a-ICS method requires no other instruments except metal baths, and the RPA-CRISPR/Cas12a-ICS platform can be performed without instruments. The RPA and enzyme digestion processes can be carried out without laboratory conditions by holding the reaction tube by hand to provide temperature, and the results can be directly observed with the naked eye. This allows for visualization and device independence. Due to its portability and ease of operation, the system could be used for on-site inspection. The LAMP, RPA-CRISPR/Cas12a-flu detection system could meet the need for higher-sensitivity detection.

Therefore, the thermostatic amplification-CRISPR/Cas12a detection platform could be combined with a variety of biosensors for the detection of foodborne pathogens or viruses, transgenic crops, etc.

## 5. Conclusions

*S. aureus* is one of the main pathogenic bacteria causing global foodborne disease outbreaks. In this study, the LAMP, RPA-CRISPR/Cas12a platform for the detection of *S. aureus* with high sensitivity, high specificity, visualization and device independence was successfully constructed. For the RPA-CRISPR/Cas12a-flu platform, low concentrations of 5.78 fg/µL and 67 CFU/mL can be detected for the *nuc* gene and *S. aureus,* respectively. The LAMP, RPA-CRISPR/Cas12a-ICS platform does not require special equipment during the inspection process and can be visually detected within 30 min. Therefore, the LAMP, RPA-CRISPR/Cas12a detection platform can meet the needs of POCT detection of *S. aureus*, which is of great significance for food safety supervision and clinical diagnosis. More importantly, it can be used not only for the detection of *S. aureus*, but also for other pathogens and viruses based on different target crRNA.

## Data Availability

The raw data supporting the conclusions of this article will be made available by the authors, without undue reservation, to any qualified researcher.

## Supplementary Materials

The following supporting information can be downloaded at: https://www.mdpi.com/article/10.3390/foods12244432/s1.
