# Supplementary material for: Isothermal Amplification and CRISPR/Cas12a-System-Based Assay for Rapid, Sensitive and Visual Detection of Staphylococcus aureus
Source: Foods. 2023 Dec 11;12(24):4432. doi: 10.3390/foods12244432 (PMC10742561; doi:10.3390/foods12244432)
Supplement: Supplementary file 1 [file foods-12-04432-s001.zip › FOODs-supplementary.pdf]

Table S1. Strain information and culture conditions for specific evaluation of the LAMP, RPA-CRISPR/cas12a platform

No.	Bacterial species	Strains	Amount of template DNA (ng/ μ L)	CRISPR/Cas12 platform results	Culture medium	Cultivation temperature	Source*
1	*S. aureus*	ATCC6538	10	+	TSB	37℃	a
2	*S. aureus*	ATCC43300	10	+	TSB	37℃	a
3	*S. xylosus*	ATCC29971	10	-	LB	37℃	b
4	*S. mimicus*	ATCC27851	10	-	LB	37℃	b
5	*E. coli.* O157:H7	ATCC43888	10	-	LB	37℃	c
6	*E. coli.* O157:H7	ATCC43895	10	-	LB	37℃	c
7	*E. coli.*	ATCC25922	10	-	LB	37℃	b
8	*E. coli.*	ATCC8739	10	-	LB	37℃	b
9	*E. coli.*	ATCC8099	10	-	LB	37℃	b
10	*V. parahaemolyti-cus*	ATCC33847	10	-	BHI	37℃	c
11	*V. parahaemolyti-cus*	ATCC17802	10	-	BHI	37℃	c
12	*V. vulnificus*	ATCC27562	10	-	BHI	30℃	c
13	*V. algolyticus*	ATCC33787	10	-	BHI	37℃	c

*a, Guangdong Microbial Culture Collection Center; b, Baosai Biotechnology Co., Ltd; c, Beijing Beinachuang Biotechnology Research Institute; TSB, Tryptic Soy Polmyxin Brot Base; LB, Luria-Bertani; BHI, Brain Heart Infusion.

Table S2. Sequences of LAMP primers, RPA primers, CrRNAs, ssDNA reporters and target DNA.

Primer name	Sequences (5′–3′)	Amplicon length (bp)
Nuc-PCR-F	AGGGCAATACGCAAAGAGGT	20
Nuc-PCR-R	GCACAAGCAAAAAAAGAGAAA	21
LAMP-F3-211bp	TCAACCAATGACATTCAGAC	20
LAMP-B3-211bp	TTACCATTTTTCCATCAGCAT	21
LAMP-FIP-211bp	TCAGGACCATATTTCTCTACACCT-TATTATTGGTT-GATACACCTGAAAC	49
LAMP-BIP-211bp	AATTGAAGTCGAGTTTGACAAAGGT-AAATATAC-GCTAAGCCACGT	45
LAMP-F3-221bp	GCATTTACGAAAAAAATGGTAGA	23
LAMP-B3-221bp	TTTTGCTTGTGCTTCACTT	19
LAMP-FIP-221bp	GCCACGTCCATATTTATCAGTTCT-AAATGCAAA-GAAAATTGAAGTCG	48
LAMP-BIP-221bp	AGCTTTAGTTCGTCAAGGCTTG-TGTTCATGTG-TATTGTTAGGTT	45
LAMP-F3-224bp	GTCAAAGAACTGATAAATATGGAC	24

LAMP-B3-224bp	ACAATGAGCATTATTGACCTG	21
LAMP-FIP-224bp	GCCAAGCCTTGACGAACTAA-GTGGCTTAGCG-TATATTTATGC	42
LAMP-BIP-224bp	TTAAGAAAAAGTGAAGCACAAGCAA-AATCAGCGTT-GTCTTCGC	43
LAMP-F3-235bp	AGCATCCTAAAAAAGGTGTAG	21
LAMP-B3-235bp	TGTTCATGTGTATTGTTAGGTT	22
LAMP-FIP-235bp	CCTTTGTCAAACTCGACTTCAATTT-AA-TATGGTCCTGAAGCAAGT	45
LAMP-BIP-235bp	TATGGACGTGGCTTAGCGTA-ACTTTAGCCAA-GCCTTGA	38
RPA-F-168bp	GGTTGATACACCTGAAACAAAGCATCCTAA	30
RPA-R-168bp	ATATGGACGTGGCTTAGCGTATATTTATGC	30
RPA-F-408bp	CTTATAGGGATGGCTATCAGTAATGTTTCG	30
RPA-R-408bp	GCATAAATATACGCTAAGCCACGTCCATAT	30
RPA-F-298bp	ATATGGTCCTGAAGCAAGTGCATTTACGAA	30
RPA-R-298bp	TGAGCATTATTGACCTGAATCAGCGTTGTC	30
RPA-F-332bp	GTTGATACACCTGAAACAAAGCATCCTAAA	30
RPA-R-332bp	ACAATGAGCATTATTGACCTGAATCAGCGT	30
crRNA-LAMP-1	AAUUUCUACUGUUGUAGAUUGCUGAUG-GAAAAAUGGUAAACG	42
crRNA-LAMP-2	AAUUUCUACUGUUGUAGAUUAAACCU-AACAAUACACAUGAAC	42
crRNA-LAMP-3	AAUUUCUACUGUUGUAGAUCGAAAAAAAUGGUA-GAAAAUGCA	42
crRNA-RPA-1	AAUUUCUACUGUUGUAGAUGCCAAGCCUUGAC-GAACUAAAGC	42
crRNA-RPA-2	AAUUUCUACUGUUGUAGAUAGAAAAAGUGAA-GCACAAGCAAA	42
flu-ssDNA	5'-6-HEX-TTTTTTTTTT-BHQ1-3'	10

ICS-ssDNA	5'-6FITC-TTATT-6-Biotin-3'	5
Target sequences	TCAACCAATGACATTCAGACTATTATTGGTTGATA-CACCTGAAACAAA-GCATCCTAAAAAAGGTGTAGAGAAATATGGTCCTGAAGCAAGTGCATTTACGAAAAAAATGG-TAGAAAATGCAAAGAAAATTGAAGTCGAGTTT-GACAAAGGTCAAAGAACTGATAAATATGGACGTGGCTTAGCGTATATTTATGCTGATGGAAAAATGGTAAAC-GAAGCTTTAGTTCGTCAAGGCTT-GGCTAAAGTTGCTTATGTTTATAAACCTAACAATACACATGAACAACTTTTAAGAAAAAGTGAAGCACAA-GCAAAAAAAGAGAAATTAAATATTT-GGAGCGAAGACAACGCTGATTCAGGTCAATAATGCTCA	370

Table S3. The specific fluorescence values of crRNA in Figure 2.

Number	Name	Fluorescence intensity (A.U)	Ratio to negative control
Fig.2A3	crRNA-1	39837.67	13.32364
	N1	2990	/
	crRNA-2	37114.33	11.66871
	N2	3180.67	/
Fig.2B3	crRNA-1	48965.33	15.57421
	N1	3144	/
	crRNA-2	43728.33	18.59198
	N2	2352	/
	crRNA-3	33979.67	13.24227
	N3	2566	/

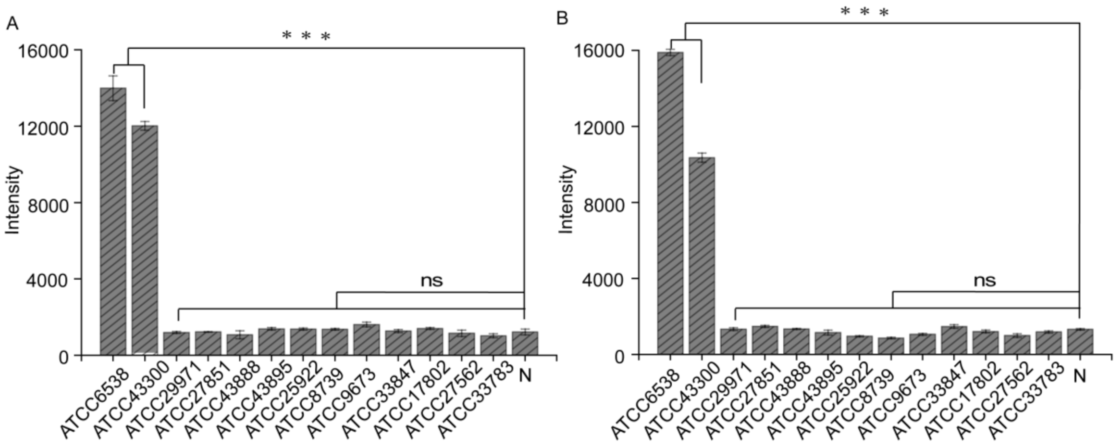

Figure S1. A. ImagJ analysis was performed on LAMP-CRISPR/Cas12a-ICS platform. B. ImagJ analysis was performed on RPA-CRISPR/Cas12a-ICS platform. (Three biological replicates) based platform with a microplate reader ($P < 0.05$). N, no template added as negative control. ***, $p < 0.001$.

Table S4. Information on 20 natural samples

No.	Name	Source*	Time
1	Chicken	a	20230412
2	fish	a	20230412
3	egg	a	20230412
4	shrimp	a	20230412
5	milk	b	20230310
6	milk	a	20230412
7	Eight treasure congee (YinLu)	c	20230412
8	soy sauce (HaiTian)	c	20230412
9	dried noodles	a	20230412
10	Cheese (Yili)	c	20230412
11	egg	b	20230310
12	beef	a	20230412
13	pork	a	20230412
14	Chiba tofu	a	20230412
15	fried tofu	a	20230412
16	tofu	a	20230412
17	fish	b	20230310
18	yogurt	a	20230412
19	salmon	a	20230412
20	duck egg	a	20230412

*a, Shanghai agricultural and sideline products wholesale market; b, Shanghai Center of Agri-Products Quality And Safety, Shanghai 200335, China; c, Ru Hai supermarket, Shanghai, China.

Table S5. Names and accession numbers of 32 strains of C. aureus retrieved from the NCBI.

No.	Name	Accession number
1	ATCC BAA-39	CP033505.1

2	ATCC BAA-39	CP033506.1
3	B119	CP038460.1
4	Be62	CP012013.1
5	Gv51	CP012015.1
6	Gv69	CP009681.1
7	Gv88	CP012018.1
8	HC1335	CP012012.1
9	HC1340	CP012011.1
10	JICS127	AP025693.1
11	JP080	AP017922.1
12	KUH140013	AP020311.1
13	KUH140046	AP020313.1
14	KUH180038	AP020318.1
15	KUH180062	AP020320.1
16	KUH180129	AP020322.1
17	KUN1163	AP020324.1
18	KUH140331	AP020316.1
19	MRSA252	BX571856.1
20	N315	BA000018.3
21	NCTC 8325	NC_007795.1
22	O17	CP032051.1
23	B118	CP038268.1
24	O82	CP038819.1
25	O217	CP038461.1
26	O267	CP034102.1
27	O268	CP038612.1
28	O326	CP032481.1
29	B114	CP038269.1
30	B115	CP038270.1
31	USA300	CP092052.1
32	USA600	CP092055.1
